# Association of plasma neutrophil gelatinase-associated lipocalin and thoracic aorta calcification in maintenance hemodialysis patients with and without diabetes

**DOI:** 10.1186/s12882-022-02773-z

**Published:** 2022-04-22

**Authors:** Kai Wei, Gesheng Song, Linhe Xi, Juan Chen, Chuancai Sun, Ping Chen, Yong Wei, Li Wang, Xianglei Kong, Yang Li, Dongmei Xu, Xiaoyan Jia

**Affiliations:** 1 Department of Nephrology, The First Affiliated Hospital of Shandong First Medical University (Shandong Provincial Qianfoshan Hospital), No.16766, Jingshi Road, Jinan, 250014 China; 2 Shandong Provincial Insititute of Nephrology, Jinan, China; 3 Department of Radiology, The First Affiliated Hospital of Shandong First Medical University (Shandong Provincial Qianfoshan Hospital), Jinan, China; 4 Department of Plastic and Reconstruction, The First Affiliated Hospital of Shandong First Medical University (Shandong Provincial Qianfoshan Hospital), Jinan, China

**Keywords:** Neutrophil gelatinase-associated lipocalin, Thoracic aorta calcification, Maintenance hemodialysis, Diabetes

## Abstract

**Background:**

Neutrophil gelatinase-associated lipocalin (NGAL) is not only a bone-derived factor involved in metabolism, but also a biomarker of kidney disease and cardiovascular pathophysiology. We conducted this cross-sectional observational study to explore relationships between plasma NGAL and thoracic aorta calcification (TAC) in maintenance hemodialysis (MHD) patients with and without diabetes.

**Methods:**

Plasma NGAL was measured by ELISA, TAC was evaluated via computed tomography scan using a 3D quantification method or chest radiography aortic arch calcification score. Spearman correlation, Logistic regression and Partial correlation analysis were used to describe the correlations between NGAL and TAC.

**Results:**

Plasma NGAL levels were lower in MHD patients with diabetes compared to those without diabetes (49.33(42.37, 55.48) vs 56.78(44.37, 674.13) ng/mL, *P* = 0.026). In MHD patients without diabetes, lg (NGAL) was positively correlated with ARC value(*R* = 0.612, *P* = 0.003) analyzed by Spearman correlation; for partial correlation analysis, lg (NGAL) was positively correlated with ARC value, after adjusting for age and sex (*R* = 0.550, *P* = 0.015), adjusting for age, sex and CHD (*R* = 0.565, *P* = 0.015), adjusting for age, sex, CHD and Alb (*R* = 0.536, *P* = 0.027), or adjusting for age, sex, CHD, Alb, and dialyzer membrane (polysulfone) (*R* = 0.590, *P* = 0.016); however, when adjusting for age, sex, CHD, Alb and Ca, the correlation between lg (NGAL) and ARC value disappeared. Positive correlation were found between NGAL and Ca (*R* = 0.644, *P* < 0.001), Ca and ACR (*R* = 0.534, *P* = 0.013) in Spearman coefficient analysis.

**Conclusion:**

There were positive correlations among plasma NGAL, serum Ca and ARC in MHD patients without diabetes; which suggests that NGAL is possibly a participant in cardiovascular calcification, in non-diabetic MHD.

**Supplementary Information:**

The online version contains supplementary material available at 10.1186/s12882-022-02773-z.

## Background

NGAL, also known as 24p3 or lipocalin2, is a 25-kDa secretory protein, which has been found to be involved in numerous functions such as bacteriostasis, iron trafficking, metabolic regulation, chemotaxis, differentiation, proliferation and so on [1–4]. Under basal conditions, NGAL is mainly expressed by osteoblasts and has been identified as a bone-derived hormone involved in appetite control and metabolism [5]. In pathological states, the expression of NGAL can be induced in many other cells and tissues [6, 7], including neutrophil granules [8], macrophages [9], dendritic cells [10], adipocytes [6, 11], cardiomyocytes [12], smooth muscle cells [7], neurons [13], liver [14] and so on. Evidence from both mouse and human studies demonstrates that NGAL is highly expressed in damaged kidney tubule cells, and it is widely accepted as one of the optimal biomarkers for kidney injury [15–17]. Moreover, NGAL is also a biomarker for cardiovascular disease [18], such as heart failure, coronary artery disease (CAD), abdominal aortic aneurysm (AAA). Our previous study [19] has demonstrated that “plasma NGAL is positively associated with parameters of CKD-MBD such as calcium (Ca), alkaline phosphatase (ALP) in maintenance hemodialysis patients (MHD)”. However, few data are available on the possible relationship between NGAL and vascular calcification, which is a common and intractable character of CKD-MBD; so we conducted this study to explore the association between plasma NGAL and thoracic aorta calcification in MHD patients. Considering that lots of studies have suggested that NGAL is closely related to diabetes and insulin resistance [18], patients were divided to 2 groups according to with or without diabetes for subgroup analysis.

## Methods

### Study population and data source

Stable adult MHD patients with available plasma samples and chest radiography or computed tomography (CT) in our center from July 2019 to December 2020 were included in the cross-sectional observational study. Inclusion criteria were age ≥ 18 years and underwent MHD for at least 3 months; exclusion criteria were Kt/V < 1.2, hospitalization within 3 months before enrollment, cancer, active inflammation, or high serum C-reactive protein. This study was approved by the Ethics Committee of the First Affiliated Hospital of Shandong First Medical University, and all subjects gave their informed consent.

Demographic and clinical characteristics were collected from medical records as we have described before [19]. In detail, demographic factors (age, sex, vintage); smoking, residual urine volume, blood pressure, comorbid coronary heart disease (CHD) or not,comorbid diabetes or not; serum Ca, phosphate (P), iPTH, ALP, potassium(K), carbonate ion (HCO3-), urea nitrogen (BUN), creatinine (Cr), albumin (Alb), low density lipoprotein (LDL), triglyceride (TG) and ferritin (SF); blood hemoglobin (Hb), white blood cells (WBC) and platelet (PLT). Prescription drugs for CKD-MBD and arteriosclerosis were also collected, including calcium free phosphorus binders (sevelamer or lanthanum carbonate), calciums, active vitamin D, paricalcitol and statins.

### Detection of plasma NGAL and klotho

Blood samples were collected and detected as previously described [19]. Blood samples were drawn from the dialysis pipeline immediately after initiation of single hemodialysis treatment. The plasma were multiple aliquoted after centrifugation and stored at − 80 °C until use. The concentrations of NGAL and Klotho were measured by ELISA Kit (Sandwich-ELISA Systems, Elabscience, China) with intra-assay and inter-assay CVs of less than 5 and 8%, respectively.

### Assessment of thoracic aorta calcification

Thoracic aorta calcification (TAC) was evaluated via chest computed tomography (CT) or routine chest radiography [20–22]. Thoracic aorta calcium was estimated by CT in mediastinal window with slice is 1.25 mm: calcification volume was quantified by extracting the aorta calcium with density ≥ 130 HU from the volume-rendered image under the assistance of semi-automatic software; thoracic aorta was divided into 4 sites, aortic root calcium (ARC), ascending aorta calcium (ATAC), aortic arch calcium (AAC), descending thoracic aorta calcium (DTAC) and total calcium (TC) were all calculated. AAC by routine chest radiography: the circumferences of aortic arch was divided into 16 sites, the number of sectors with calcification was counted and divided by 100 to express the results as a percentage.

### Statistical analyses

Categorical data are presented as frequencies and percentages. Continuous variables are expressed as mean ± standard deviation (SD) for normally distributed variables and as median (interquartile range [IQR]) for variables with skewed distribution. Patients were subsequently divided into 2 groups according to comorbid diabetes or not for subgroup analysis. Differences between patients with and without diabetes were assessed using the t test, Mann–Whitney U test or Chi-square analysis, as appropriate. Spearman correlation coefficients, Logistic regression analyses and Partial correlation analysis were used to describe the correlations between NGAL and TAC. All statistical tests were two-sided, a *P*-value of less than 0.05 was considered as statistically significant. All statistical analyses were performed using SPSS 20.0 (SPSS, Chicago, IL).

## Results

### Demographic, clinical characteristics, NGAL and TAC in all MHD patients

A total of 62 stable adult MHD patients were finally included in the cross-sectional observational study (in Table 1). All patients received the same dialysis modality, that is three sessions per week, for 4 h per session. The dialysate calcium concentration = 1.25 mmol/L for patients with adjusted serum calcium> 2.5 mmol/L or confirmed ectopic calcification, and dialysate calcium concentration = 1.5 mmol/L for other patients. The average age was 58.10 ± 15.98 years; male to female ratio was 1:0.94; median vintage was 29.27(8.65, 78.02) months; 43.55% of patients comorbid diabetes, 30.6% comorbid CHD. The median concentration of NGAL was 52.02(43.11, 422.30) ng/mL. As shown in the Table 2, there were 43 patients with chest CT, the TAC incidences were: TC 81.4%, ARC 30.2%, ATAC 34.9%, AAC 81.4%, DTAC 74.4%; 24 patients with routine chest radiography, the AAC incidence was 75%; 49 cases with AAC estimated by CT or routine chest radiography, the AAC incidence was 79%. Table 3 shows the values of TAC of 43 patients with chest CT: TC 9.55(0.70, 23.65) mL, ARC 0(0, 0.46) mL, ATAC 0(0, 0.58) mL, AAC 3.65(0.49, 8.08) mL, DTAC5.38(0, 11.71) mL; AAC values of 24 patients with routine chest radiography was 21.88(1.56, 35.94)%.

**Table 1:** Characteristics of total MHD patients, MHD patients with and without diabetes

Characteristic	Total(*N* = 62)	With diabetes(*N* = 27)	Without diabetes(*N* = 35)	P
Male(0)/female(1)	30/32	14/13	16/19	0.798
Age (year)	58.10 ± 15.98	65.33 ± 13.17	52.51 ± 15.87	**0.001**
Smoking, (%)	21.0	25.9	17.1	0.297
Vintage (months)	29.27(8.65, 78.02)	19.28(3.78, 52.50)	40.27(16.16, 98.00)	**0.007**
Residual urine volume (ml/24 h)	0(0, 200)	100(0, 400)	0(0, 150)	0.123
SBP (mmHg)	155.05 ± 21.53	163.37 ± 17.68	148.63 ± 22.24	**0.005**
DBP (mmHg)	79.03 ± 16.13	68.48 ± 11.92	87.17 ± 14.32	**< 0.001**
Comorbid CHD, (%)	30.6	40.7	22.9	0.108
NGAL (ng/mL)	52.02(43.11, 422.30)	49.33(42.37,55.48)	56.78(44.37,674.13)	**0.026**
Klotho (pg/mL)	308.49(76.94, 877.32)	265.22(11.70, 1391.19)	319.96(138.46,608.23)	0.836
Ca (mmol/L)	2.29(2.17, 2.41)	2.21(2.12, 2.32)	2.35(2.27, 2.51)	**0.001**
P (mmol/L)	1.82(1.52, 2.26)	1.67(1.49, 1.91)	1.97(1.53, 2.53)	**0.039**
iPTH (pg/mL)	245.4 0(122.35, 864.75)	165.00(79.53, 274.80)	745.60(162.30, 1775.00)	**< 0.001**
ALP(U/L)	75.00(60.00, 146.25)	73.00(59.00, 91.00)	110.00(60.00, 353.00)	**0.019**
K (mmol/L)	5.02(4.44, 5.51)	4.86(4.45, 5.73)	5.10(4.40, 5.45)	0.943
HCO3^−^(mmol/L)	20.04 ± 3.25	20.21 ± 2.90	19.91 ± 3.53	0.716
BUN (mmol/L)	24.66 ± 6.09	23.50 ± 5.79	25.55 ± 6.24	0.191
Cr (μmol/L)	786.50(680.60, 922.00)	707.00(535.00, 752.00)	884.00(754.00, 1038.00)	**< 0.001**
Alb(g/L)	41.1(39.0, 43.75)	39.30(35.30, 42.10)	42.1(39.8, 44.60)	**0.005**
LDL (mmol/L)	2.04 ± 0.59	2.02 ± 0.67	2.06 ± 0.52	0.800
TG (mmol/L)	1.22(0.88,1.77)	1.12(0.88,1.75)	1.29(0.88,2.17)	0.247
SF (ng/mL)	155.10(80.13, 317.25)	135.87(83.65, 249.71)	197.64(78.19, 405.49)	0.089
Hb(g/L)	112.00(100.00, 121.25)	117.00(100.00, 124.00)	111.00(100.00, 119.00)	0.259
WBC(910^9/L)	6.06(4.55,7.37)	6.62(5.47, 7.98)	5.11(4.23, 7.29)	**0.025**
PLT(510^9/L)	189.50(160.00, 22D1.75)	203.00(145.00, 247.00)	184.00(164.00, 199.00)	0.338
Dialyzer membrane				
CTA, (%)	35.5	22.2	45.7	**0.048**
PS, (%)	54.8	63.0	48.6	0.192
PMMA, (%)	9.7	14.8	5.7	0.221
Calciums, (%)	79.0	66.7	88.6	**0.037**
Calcium free phosphorus binders carbonate)				
Sevelamer, (%)	25.8	3.7	42.9	**< 0.001**
Lanthanum carbonate, (%)	16.1	18.5	14.3	0.735
Active vitamin D, (%)	29.0	7.4	45.7	**0.001**
Paricalcitol, (%)	8.1	3.7	11.4	0.376
Statins, (%)	16.1	33.3	2.9	**0.002**

Continuous variables are expressed as mean ± standard deviation for normally distributed variables and as median (interquartile range) for variables with skewed distribution. Differences between patients with and without diabetes were assessed using the t test, Mann–Whitney U test or Chi-square analysis, as appropriate. Values with *p* < 0.05 were considered statistically significant and are indicated in bold

*MHD* maintenance hemodialysis, *SBP* systolic blood pressure, *DBP* diastolic blood pressure, *CHD* Coronary heart disease, *NGAL* neutrophil gelatinase-associated lipocalin, *Ca* calcium, *P* phosphate, *iPTH* intact parathyroid hormone, *ALP* alkaline phosphatase, *K* potassium, *HCO3*- carbonate ion, *BUN* blood urea nitrogen, *Cr* creatinine, *Alb* albumin, *LDL* low density lipoprotein, *TG* triglyceride, *SF* serum ferritin, *Hb* hemoglobin, *WBC* white blood cells, *PLT* platelet, *HD* hemodialysis, *HFD* high-flux hemodialysis, *HDF* hemodiafiltration, *CTA* cellulose triacetate, *PS* polysulfone, *PMMA* polymethylmethacrylate

**Table 2:** Thoracic aorta calcification incidences of total MHD patients, MHD patients with and without diabetes

Characteristic	Total (*N* = 62)	With diabetes (*N* = 27)	Without diabetes (*N* = 35)	P
CT	*N* = 43	*N* = 22	*N* = 21	
TC	35, 81.4%	19, 86.4%	16, 76.2%	0.457
ARC	13, 30.2%	7, 31.8%	6, 28.6%	1.000
ATAC	15, 34.9%	8, 36.8%	7, 33.3%	1.000
AAC	35, 81.4%	19, 86.4%	16, 76.2%	0.457
DTAC	32, 74.4%	18, 81.8%	14, 66.7%	0.310
Routine chest radiography	*N* = 24	*N* = 6	*N* = 18	
AAC	18, 75%	6, 100%	12, 66.7%	0.277
AAC by CT or chest radiography	*N* = 62	*N* = 27	*N* = 35	
	49, 79.0%	24, 88.9%	25, 71.4%	0.122

Thoracic aorta calcium estimated by computed tomography (CT): calcification volume quantified by extracting the aorta calcium with density ≥ 130 HU from the volume-rendered image under the assistance of semi-automatic software; thoracic aorta was divided into 4 sites, aortic root calcium (ARC), ascending aorta calcium (ATAC), AAC (aortic arch calcium), DTAC(descending thoracic aorta calcium) and TC (total calcium) were all calculated

AAC by routine chest radiography: the circumferences of aortic arch was divided into 16 sites, the number of sectors with calcification was calculated and multiplied by 100 to express the results as a percentage

**Table 3:** Thoracic aorta calcification parameter values of total MHD patients, MHD patients with and without diabetes

Characteristic	Total (*N* = 62)	With diabetes (*N* = 27)	Without diabetes (*N* = 35)	P
CT	*N* = 43	*N* = 22	*N* = 21	
TC (mL)	9.55(0.70, 23.65)	11.86(4.74, 32.09)	5.27(0.14, 18.13)	0.176
ARC (mL)	0(0, 0.46)	0(0, 0.65)	0(0, 0.39)	0.709
ATAC (mL)	0(0, 0.58)	0(0, 0.69)	0(0, 0.64)	0.819
AAC (mL)	3.65(0.49, 8.08)	4.32(0.61, 8.28)	1.70(0.14, 9.21)	0.670
DTAC (mL)	5.38(0, 11.71)	7.05(2.24, 20.29)	3.31(0, 8.85)	0.070
Routine chest radiography	*N* = 24	*N* = 6	*N* = 18	
AAC(%)	21.88(1.56, 35.94)	31.25(25.00, 53.12)	18.75(0, 28.13)	0.090

Thoracic aorta calcium estimated by computed tomography (CT): calcification volume quantified by extracting the aorta calcium with density ≥ 130 HU from the volume-rendered image under the assistance of semi-automatic software; thoracic aorta was divided into 4 sites, aortic root calcium (ARC), ascending aorta calcium (ATAC), AAC(aortic arch calcium), DTAC(descending thoracic aorta calcium) and TC (total calcium) were all calculated

AAC by routine chest radiography: the circumferences of aortic arch was divided into 16 sites, the number of sectors with calcification was calculated and multiplied by 100 to express the results as a percentage

### Differences of demographic, clinical characteristics, NGAL and TAC between patients with and without diabetes

Characteristics of study participants are shown in Table 1. Compared with patients without diabetes, patients with diabetes were significantly older (65.33±13.17 vs 52.51±15.87 years, *P* = 0.001); and with shorter vintage (19.28(3.78, 52.50) vs 40.27(16.16, 98.00) months, *P* = 0.007), higher systolic blood pressure (SBP) (163.37 ± 17.68 vs 148.63 ± 22.24 mmHg, *P* = 0.005), lower diastolic blood pressure (DBP) (68.48 ± 11.92 vs 87.17 ± 14.32 mmHg, *P* < 0.001), lower plasma NGAL(49.33(42.37,55.48) vs 56.78(44.37,674.13) ng/mL, *P* = 0.026), lower Ca(2.21(2.12, 2.32) vs 2.35 (2.27, 2.51) mmol/L, *P* = 0.001), lower P (1.67(1.49, 1.91) vs 1.97(1.53, 2.53) mmol/L, *P* = 0.039), lower iPTH (165.00(79.53, 274.80) vs 745.60(162.30, 1775.00)pg/mL, *P* < 0.001), lower ALP(73.00(59.00, 91.00) vs 110.00(60.00, 353.00)U/L, *P* = 0.019), lower Cr(707.00(535.00, 752.00) vs 884.00(754.00, 1038.00) 38.00) *P* < 0.001), lower Alb(39.30(35.30, 42.10) vs 42.1(39.8, 44.60) g/L, *P* = 0.005), higher WBC(6.62(5.47, 7.98) vs5.11(4.23, 7.29) × 10^9/L, *P* = 0.025). In terms of the application of medicine, lesser proportion of patients with diabetes taking calcium (66.7% vs 88.6%, *P* = 0.037), sevelamer(3.7% vs 42.9%), active vitamin D(7.4% vs 45.7%, *P* = 0.001), while larger proportion of them taking statins(33.3% vs 2.9%, *P* = 0.002). All estimated TAC parameters incidences (Table 2) or values (Table 3) were higher in MHD patients with diabetes than those without diabetes; however, the differences were not statistically significant.

### Correlation analysis between plasma NGAL and TAC

Univariable logistic regression analyses showed positive correlations between lg (NGAL) and ARC (Table 4, OR = 5.581, *P* = 0.028), no correlations were found between lg (NGAL) and other TAC parameters. Age, sex and positive factors calculated by univariable logistic regression analyses (supplemental Table 1) between ARC and all characters were used for multivariable models (Table 5); lg (NGAL) was positively correlated with ARC after adjusting for age and sex (OR = 13.335, *P* = 0.012), adjusting for CHD (OR = 18.675, *P* = 0.014), or adjusting for active vitamin D (OR = 4.988, *P* = 0.043); however, when adjusting for Ca or vintage, the correlation between lg (NGAL) and ARC disappeared.

**Table 4:** Univariable logistic regression analyses of lg (NGAL (ng/mL)) and thoracic aorta calcification in MHD patients (*n* = 62)

Characteristic	OR (95% CI)	P
CT	*N* = 43	
TC	1.124(0.213-5.946)	0.890
ARC	5.581(1.204-25.871)	**0.028**
ATAC	1.586(0.431-5.834)	0.488
AAC	1.124(0.213-5.946)	0.890
DTAC	1.539(0.317-7.468)	0.593
Routine chest radiography	*N* = 24	
AAC	0.499(0.101-2.470)	0.394
AAC by CT or chest radiography	*N* = 62	
	0.561(0.195-1.611)	0.283

Thoracic aorta calcium estimated by computed tomography (CT): calcification volume quantified by extracting the aorta calcium with density ≥ 130 HU from the volume-rendered image under the assistance of semi-automatic software; thoracic aorta was divided into 4 sites, aortic root calcium (ARC), ascending aorta calcium (ATAC), AAC(aortic arch calcium), DTAC(descending thoracic aorta calcium) and TC (total calcium) were all calculated

AAC by routine chest radiography: the circumferences of aortic arch was divided into 16 sites, the number of sectors with calcification was calculated and multiplied by 100 to express the results as a percentage

**Table 5:** Logistic regression analyses of lg (NGAL (ng/mL)) and aortic root calcium (ARC) in MHD patients (*n* = 43)

Models	OR (95% CI)	P
Univariable analysis	5.581(1.204-25.871)	**0.028**
Multivariable-adjusted analysis		
Age and sex	13.335(1.781-99.829)	**0.012**
Comorbid CHD	18.675(1.791-194.711)	**0.014**
Ca	2.561(0.342-19.172)	0.360
Vintage (months)	3.410(0.497-23.393)	0.212
Active vitamin D	4.988(1.053-23.641)	**0.043**

*NGAL* neutrophil gelatinase-associated lipocalin, *CHD* Coronary heart disease, *Ca* calcium

**Fig. 1:**
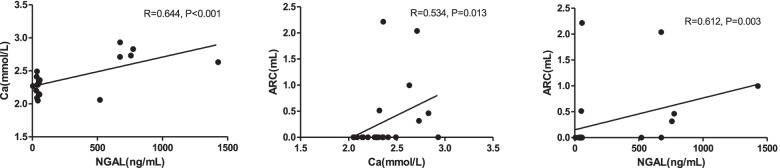
Scatter diagrams and Spearman coefficient between NGAL, Ca and ARC of MHD patients without diabetes (*n* = 21)

Spearman correlation analysis between lg (NGAL) and TAC parameter values are depicted in Table 6. In MHD patients without diabetes (*n* = 21), lg (NGAL) were positively correlated with ARC value (*R* = 0.612, *P* = 0.003); no other correlations were found in total group or subgroups. Spearman correlation analysis between ARC value and all demographic, clinical characters were further performed in without diabetes group (supplemental Table 2); and 4 potential confounders were found as follows, comorbid CHD (*R* = 0.481, *P* = 0.027), Ca (mmol/L)(*R* = 0.534, *P* = 0.013), Alb(g/L)(*R* = -0.539, *P* = 0.012), dalyzer membrane (polysulfone)(*R* = -0.458, *P* = 0.037). Age, sex and all confounders were employed in partial correlation analysis in patients without diabetes (Table 7). Lg (NGAL) was positively correlated with ARC value, after adjusting for age and sex(*R* = 0.550, *P* = 0.015), adjusting for age, sex and CHD(*R* = 0.565, *P* = 0.015), adjusting for age, sex, CHD and Alb(*R* = 0.536, *P* = 0.027), or adjusting for age, sex, CHD, Alb, and dialyzer membrane (polysulfone)(*R* = 0.590, *P* = 0.016); however, when adjusting for age, sex, CHD, Alb, and Ca, the correlation between lg (NGAL) and ARC value disappeared. Scatter diagrams and Spearman coefficient are shown in Fig. 1, positive correlation were found between NGAL and Ca(*R* = 0.644, *P* < 0.001), Ca and ARC(*R* = 0.534, *P* = 0.013), NGAL and ACR(*R* = 0.612, *P* = 0.003).

**Table 6:** Spearman coefficients between lg (NGAL (ng/mL)) and thoracic aorta calcification parameter values of total MHD patients, MHD patients with and without diabetes

Characteristic	Total			With diabetes			Without diabetes		
CT	*N* = 43	R	P	*N* = 22	R	P	*N* = 21	R	P
TC (mL)		0.081	0.607		−0.025	0.911		0.149	0.519
ARC (mL)		0.271	0.079		−0.076	0.737		**0.612**	**0.003**
ATAC (mL)		0.092	0.555		−0.127	0.573		0.273	0.231
AAC (mL)		0.107	0.495		0.143	0.525		0.068	0.770
DTAC (mL)		0.060	0.702		−0.113	0.618		0.169	0.465
Routine chest radiography									
AAC(%)	*N* = 24	−0.199	0.351	*N* = 6	0.213	0.686	*N* = 18	−0.053	0.835

Thoracic aorta calcium estimated by computed tomography (CT): calcification volume quantified by extracting the aorta calcium with density and without diabetesiplied by 100 to express the results as a percentage.matic software; thoracic aorta was divided into 4 sites, aortic root calcium (ARC), ascending aorta calcium (ATAC), AAC(aortic arch calcium), DTAC(descending thoracic aorta calcium) and TC (total calcium) were all calculated

AAC by routine chest radiography: the circumferences of aortic arch was divided into 16 sites, the number of sectors with calcification was calculated and multiplied by 100 to express the results as a percentage

**Table 7:** Partial coefficients between lg (NGAL (ng/mL)) and aortic root calcium (ARC) parameter values of MHD patients without diabetes (*n* = 21)

Adjusted characters	R	P
Age and sex	0.550	**0.015**
Age, sex and comorbid CHD	0.565	**0.015**
Age, sex, comorbid CHD and Alb	0.536	**0.027**
Age, sex, comorbid CHD, Alb, and Ca	0.175	0.518
Age, sex, comorbid CHD, Alb, and Dialyzer membrane(PS)	0.590	**0.016**

*NGAL* neutrophil gelatinase-associated lipocalin, *CHD* Coronary heart disease, *Alb* albumin, *Ca* calcium, *PS* polysulfone

## Discussion

Cardiovascular calcification is a common and refractory complication of MHD patients, and is closely related to the high cardiovascular morbidity and mortality [18, 23]. In addition to conventional risk factors such as diabetes, hypertension and lipid metabolism disorder; disturbance of mineral metabolism is also associated with cardiovascular calcification [24–26]. To assess thoracic aorta calcification through chest CT, we employed a 3D visualization and quantification method to divided thoracic aorta into 4 sites, ARC, ATAC, AAC, DTAC [22]. The final results showed that NGAL, a bone-derived factor involved in metabolism and a biomarker of kidney injury and cardiovascular pathophysiology, were positively correlated with serum Ca and ARC in MHD patients without diabetes. Previous studies have confirmed that ARC is associated with total coronary artery calcification and the number of coronary stenotic vessels in patients with an intermediate pretest probability of ischemic heart disease [27]. NGAL is also an independent factor for predicting major adverse cardiac events in familial hypercholesterolemia [28]. To our knowledge, this is the first study to find an association between NGAL and ARC in MHD patients; the result suggests that NGAL is an important link in the cross-talk between bone and vascular. Former observational studies have demonstrated that increased circulating NGAL levels are associated with the severity of coronary artery disease [29] and increased risk of future cardiovascular disease events [23, 30, 31]. Results from basic researches reveal the detrimental roles of NGAL in the development of cardiovascular pathophysiological process, such as atherosclerosis [18], abdominal aortic aneurysm [32] and endothelial dysfunction [33, 34].

In physiological condition, NGAL was once thought to be secreted mainly by adipose tissue; however recent study showed that it is “expressed by osteoblasts, at levels that are at least tenfold higher in osteoblasts than in white adipose tissue or other organs” [5]. In pathological conditions, NGAL is expressed by various injured cells and tissues. It is an excellent biomarker in acute kidney injure secreted by tubular epithelial cell. In MHD population, there is no difference in plasma NGAL in patients with or without kidneys, which suggest that kidney is not the main source of increased circulating NGAL [35]. In our former study [19], positive correlations between circulating NGAL and CKD-MBD parameters (serum ALP and Ca) were found in MHD patients; while a downward trend in plasma NGAL was observed after PTX + AT in MHD patients with severe SHPT. These results suggested that “bone maybe one of the main sources of increased NGAL in MHD patients”. In the present study, MHD patients with diabetes showed lower plasma NGAL level, the absent correlation between NGAL and ARC may due to impaired osteoblast function in diabetes [36–38]. It is confirmed that CKD patients with diabetes are more prone to present with low bone turnover states [39], the potential mechanisms probably lie in the suppression of parathyroid hormone secretion, osteoblast activity and bone turnover by hyperglycemia and insulin deficiency [40, 41].

Above all, the major finding of our study is that there were positive correlations among plasma NGAL, serum Ca and ARC in MHD patients without diabetes; which suggests that NGAL is possibly a participant in cardiovascular calcification under MHD condition. However, this is a cross-sectional observational study. Small sample size and no follow-up observations of cardiovascular event or mortality are limitations of this study and also the direction of future research.

## Conclusion

There were positive correlations among plasma NGAL, serum Ca and ARC in MHD patients without diabetes; which suggests that NGAL is possibly a participant in cardiovascular calcification, in non-diabetic MHD.

## Supplementary Information

**Additional file 1 Supplemental Table 1.** Univariable logistic regression analyses between aortic root calcium (ARC) and characters of all MHD patients (*n* = 43) MHD maintenance hemodialysis, SBP systolic blood pressure, DBP diastolic blood pressure, CHD Coronary heart disease, NGAL neutrophil gelatinase-associated lipocalin, Ca calcium, P phosphate, iPTH intact parathyroid hormone, ALP alkaline phosphatase, K potassium, HCO3- carbonate ion, BUN blood urea nitrogen, Cr creatinine, Alb albumin, LDL low density lipoprotein, TG triglyceride, SF serum ferritin; Hb hemoglobin, WBC white blood cells, PLT platelet, HD hemodialysis, HFD high-flux hemodialysis, HDF hemodiafiltration, CTA cellulose triacetate, PS polysulfone, PMMA polymethylmethacrylate. **Supplemental Table 2.** Spearman coefficients between aortic root calcium (ARC) and characters of MHD patients without diabetes (*n* = 21) MHD maintenance hemodialysis, SBP systolic blood pressure, DBP diastolic blood pressure, CHD Coronary heart disease, NGAL neutrophil gelatinase-associated lipocalin, Ca calcium, P phosphate, iPTH intact parathyroid hormone, ALP alkaline phosphatase, K potassium, HCO3- carbonate ion, BUN blood urea nitrogen, Cr creatinine, Alb albumin, LDL low density lipoprotein, TG triglyceride, SF serum ferritin; Hb hemoglobin, WBC white blood cells, PLT platelet, HD hemodialysis, HFD high-flux hemodialysis, HDF hemodiafiltration, CTA cellulose triacetate, PS polysulfone, PMMA polymethylmethacrylate.

## Data Availability

The datasets used and/or analyzed during the current study are available from the corresponding author on reasonable request.

## Abbreviations

NGAL: Neutrophil gelatinase-associated lipocalin
TAC: Thoracic aorta calcification
MHD: Maintenance hemodialysis
CAD: Coronary artery disease
AAA: Abdominal aortic aneurysm
Ca: Calcium
P: Phosphate
ALP: Alkaline phosphatase
CT: Computed tomography
CHD: Coronary heart disease
K: Potassium
HCO3-: Carbonate ion
BUN: Urea nitrogen
Cr: Creatinine
Alb: Albumin
LDL: Low density lipoprotein
TG: Triglyceride
SF: Territin
Hb: Hemoglobin
WBC: White blood cells
PLT: Platelet
ARC: Aortic root calcium
ATAC: Ascending aorta calcium
AAC: Aortic arch calcium
DTAC: Descending thoracic aorta calcium
TC: Total calcium
SD: Standard deviation
IQR: Interquartile range
SBP: Systolic blood pressure
DBP: Diastolic blood pressure
iPTH: Intact Parathyroid hormone
