# Supplementary material for: Association of plasma neutrophil gelatinase-associated lipocalin and thoracic aorta calcification in maintenance hemodialysis patients with and without diabetes
Source: BMC Nephrol. 2022 Apr 22;23:156. doi: 10.1186/s12882-022-02773-z (PMC9026670; doi:10.1186/s12882-022-02773-z)
Supplement: Supplementary file 1 — Additional file 1 Supplemental Table 1. Univariable logistic regression analyses between aortic root calcium (ARC) and characters of all MHD patients (n = 43) MHD maintenance hemodialysis, SBP systolic blood pressure, DBP diastolic blood pressure, CHD Coronary heart disease, NGAL neutrophil gelatinase-associated lipocalin, Ca calcium, P phosphate, iPTH intact parathyroid hormone, ALP alkaline phosphatase, K potassium, HCO3- carbonate ion, BUN blood urea nitrogen, Cr creatinine, Alb albumin, LDL low density lipoprotein, TG triglyceride, SF serum ferritin; Hb hemoglobin, WBC white blood cells, PLT platelet, HD hemodialysis, HFD high-flux hemodialysis, HDF hemodiafiltration, CTA cellulose triacetate, PS polysulfone, PMMA polymethylmethacrylate. Supplemental Table 2. Spearman coefficients between aortic root calcium (ARC) and characters of MHD patients without diabetes (n = 21) MHD maintenance hemodialysis, SBP systolic blood pressure, DBP diastolic blood pressure, CHD Coronary heart disease, NGAL neutrophil gelatinase-associated lipocalin, Ca calcium, P phosphate, iPTH intact parathyroid hormone, ALP alkaline phosphatase, K potassium, HCO3- carbonate ion, BUN blood urea nitrogen, Cr creatinine, Alb albumin, LDL low density lipoprotein, TG triglyceride, SF serum ferritin; Hb hemoglobin, WBC white blood cells, PLT platelet, HD hemodialysis, HFD high-flux hemodialysis, HDF hemodiafiltration, CTA cellulose triacetate, PS polysulfone, PMMA polymethylmethacrylate. [file 12882_2022_2773_MOESM1_ESM.docx]

Supplemental table1. Univariable logistic regression analyses between aortic root calcium (ARC) and characters of all MHD patients (n=43)

| Character | OR (95% CI) | P |
| --- | --- | --- |
| Male(0)/female(1) | 0.750(0.203-2.766) | 0.666 |
| Age (year) | 1.037(0.991-1.086) | 0.118 |
| Smoking | 2.889(0.595-14.021) | 0.188 |

| Vintage (months) | 1.019(1.001-1.037) | **0.037** |
| --- | --- | --- |

| Residual urine volume (ml/24h) | 0.998(0.995-1.001) | 0.998 |
| --- | --- | --- |
| SBP(mmHg) | 0.997(0.968-1.028) | 0.997 |
| DBP(mmHg) | 0.953(0.907-1.002) | 0.061 |
| Comorbid CHD | 13.333(2.774-64.089) | **<0.001** |
| Comorbid diabetes, | 0.857(0.233-3.159) | 0.857 |
| Klotho(pg/mL) | 1.0001(0.9999-1.0004) | 0.274 |
| Ca(mmol/L) | 57.233(1.680-1949.187) | **0.025** |
| P(mmol/L) | 0.887(0.558-1.411) | 0.613 |
| iPTH(pg/mL) | 1.001(0.9999-1.002) | 0.078 |
| ALP(U/L) | 1.000(0.998-1.002) | 0.931 |
| K(mmol/L) | 0.651(0.277-1.529) | 0.651 |
| HCO3-(mmol/L) | 1.059(0.875-1.283) | 0.556 |
| BUN(mmol/L) | 0.972(0.871-1.084) | 0.605 |
| Cr(μmol/L) | 0.998(0.994-1.001) | 0.146 |
| Alb(g/L) | 0.928(0.799-1.078) | 0.330 |
| LDL(mmol/L) | 1.526(0.527-4.416) | 0.436 |
| TG(mmol/L) | 1.174(0.471-2.929) | 0.731 |
| SF(ng/mL) | 1.000(0.995-1.005) | 0.945 |
| Hb(g/L) | 1.026(0.977-1.077) | 0.301 |
| WBC(×10^9/L) | 0.923(0.637-1.338) | 0.673 |
| PLT(×10^9/L) | 0.989(0.977-1.002) | 0.097 |
| Dialyzer membrane |  |  |
| CTA(ref) | (ref) | 0.581 |
| PS | 0.505(0.119-2.145) | 0.355 |
| PMMA | 1.067(0.129-8.793) | 0.952 |
| Calciums | 6.000(0.681-52.900) | 0.107 |
| Calcium free phosphorus binders carbonate) |  |  |
| No(ref) | (ref) | 0.738 |
| Sevelamer | 0.630(0.105-3.781) | 0.613 |
| Lanthanum carbonate | 0.540(0.092-3.159) | 0.494 |
| Active vitamin D | 7.714(1.533-38.829) | **0.013** |
| Paricalcitol | 0(-) | 0.999 |
| Statins | 1.200(0.250-5.768) | 0.820 |

MHD maintenance hemodialysis, SBP systolic blood pressure, DBP diastolic blood pressure, CHD Coronary heart disease, NGAL neutrophil gelatinase-associated lipocalin, Ca calcium, P phosphate, iPTH intact parathyroid hormone, ALP alkaline phosphatase, K potassium, HCO3- carbonate ion, BUN blood urea nitrogen, Cr creatinine, Alb albumin, LDL low density lipoprotein, TG triglyceride, SF serum ferritin; Hb hemoglobin, WBC white blood cells, PLT platelet, HD hemodialysis, HFD high-flux hemodialysis, HDF hemodiafiltration, CTA cellulose triacetate, PS polysulfone, PMMA polymethylmethacrylate

Supplemental table2. Spearman coefficients between aortic root calcium (ARC) and characters of MHD patients without diabetes (n=21)

| Character | R | P |
| --- | --- | --- |
| Male(0)/female(1) | 0.099 | 0.670 |
| Age (year) | 0.350 | 0.119 |
| Smoking, (%) | 0.235 | 0.305 |
| Vintage (months) | 0.279 | 0.220 |
| Residual urine volume (ml/24h) | -0.142 | 0.539 |
| SBP(mmHg) | -0.217 | 0.345 |
| DBP(mmHg) | -0.235 | 0.306 |
| Comorbid CHD, (%) | 0.481 | **0.027** |
| NGAL(ng/mL) | 0.612 | 0.003 |
| Klotho(pg/mL) | 0.146 | 0.528 |
| Ca(mmol/L) | 0.534 | **0.013** |
| P(mmol/L) | -0.038 | 0.869 |
| iPTH(pg/mL) | 0.262 | 0.251 |
| ALP(U/L) | 0.287 | 0.206 |
| K(mmol/L) | 0.094 | 0.684 |
| HCO3-(mmol/L) | 0.234 | 0.308 |
| BUN(mmol/L) | -0.151 | 0.512 |
| Cr(μmol/L) | -0.366 | 0.102 |
| Alb(g/L) | -0.539 | **0.012** |
| LDL(mmol/L) | 0.253 | 0.269 |
| TG(mmol/L) | 0.170 | 0.461 |
| SF(ng/mL) | 0.111 | 0.652 |
| Hb(g/L) | 0.026 | 0.911 |
| WBC(×10^9/L) | 0.214 | 0.351 |
| PLT(×10^9/L) | 0.035 | 0.880 |
| Dialyzer membrane |  |  |
| CTA(ref) | 0.397 | 0.074 |
| PS | -0.458 | **0.037** |
| PMMA | 0.134 | 0.562 |
| Calciums | 0.301 | 0.184 |
| Calcium free phosphorus binders carbonate |  |  |
| Sevelamer | 0.042 | 0.857 |
| Lanthanum carbonate | -0.046 | 0.842 |
| Active vitamin D | 0.397 | 0.074 |
| Paricalcitol | -0.201 | 0.381 |
| Statins | -0.139 | 0.548 |

MHD maintenance hemodialysis, SBP systolic blood pressure, DBP diastolic blood pressure, CHD Coronary heart disease, NGAL neutrophil gelatinase-associated lipocalin, Ca calcium, P phosphate, iPTH intact parathyroid hormone, ALP alkaline phosphatase, K potassium, HCO3- carbonate ion, BUN blood urea nitrogen, Cr creatinine, Alb albumin, LDL low density lipoprotein, TG triglyceride, SF serum ferritin; Hb hemoglobin, WBC white blood cells, PLT platelet, HD hemodialysis, HFD high-flux hemodialysis, HDF hemodiafiltration, CTA cellulose triacetate, PS polysulfone, PMMA polymethylmethacrylate
